# Impact of nitrogen fertilization on soil microbial diversity, its mediated enzyme activities, and stem nematode population in sweet potato fields

**DOI:** 10.3389/fmicb.2025.1528575

**Published:** 2025-04-15

**Authors:** Fengyu Shi, Xinpeng Meng, Jiaxin Li, Dan Yang, Jianbin Liu, Xingzhong Liu, Meichun Xiang, Yingbo Zhu

**Affiliations:** ^1^Hebei Key Laboratory of Crop Stress Biology, Hebei Normal University of Science and Technology, Qinhuangdao, China; ^2^Institute of Plant Nutrition, Resources and Environment, Beijing Academy of Agriculture and Forestry Sciences, Beijing, China; ^3^Department of Microbiology, College of Life Sciences, Nankai University, Tianjin, China; ^4^State Key Laboratory of Mycology, Institute of Microbiology, Chinese Academy of Sciences, Beijing, China

**Keywords:** *Ditylenchus destructor*, Illumina MiSeq sequencing, biolog, pest management, phosphatase

## Abstract

Excessive nitrogen fertilization in sweet potato cultivation poses significant ecological and economic challenges in China, negatively impacting soil health by altering microbial community diversity, enzyme activities, and increasing the risk of stem nematode damage. In this study, we conducted a field trial in Northeast China, applying 0–72 kg of urea-N per hectare to brown soil under a five-year sweet potato cropping system. The results demonstrated that optimal nitrogen fertilization (64.8 kg ha^−1^) significantly promoted beneficial microbial populations, enhanced soil urease activity, and reduced the incidence of stem nematode disease while maintaining high sweet potato yields.

## 1 Introduction

Soil microorganisms and their enzymes are essential components of soil ecosystems, playing a crucial role in organic matter decomposition and nutrient availability (Enwall et al., 2007; Bergstrom et al., 1998; Etesami, 2024). These biological factors respond rapidly to environmental changes, including soil fertilization. Enhancing soil microbial activity and enzyme functions can promote plant growth, mitigate pest and disease damage, improve soil fertility, and increase crop yields (Sparling, 1997; Belay et al., 2002; Liu et al., 2007). Numerous studies have shown that fertilization influences soil microbial biomass and alters carbon (C), nitrogen (N), and phosphorus (P) dynamics. Fertilization also regulates nutrient availability and enhances soil enzymatic activities, with urease, phosphatase, and invertase playing key roles in the N, P, and C cycles (Zhao et al., 2009). These enzymes directly participate in critical biochemical reactions, facilitating nutrient absorption by crops and contributing to soil fertility. Moreover, different fertilization levels significantly impact soil microbial communities and enzyme activities (Vepsäläinen et al., 2001).

Sweet potato [*Ipomoea batatas* (L.) Lam.] is a nutrient-rich crop, providing fiber, minerals, vitamins, and antioxidants, with low fat content (Sebben et al., 2017). Additionally, it serves as an important bioenergy resource (Lareo et al., 2013). China is the world’s largest producer of sweet potatoes, with a cultivation area of 5.5 million hectares and a total yield of 106 million metric tons (Wang et al., 2010). However, excessive fertilization and continuous cropping have led to severe issues in sweet potato production, including soil micro-ecological deterioration and an increased incidence of stem nematode (*Ditylenchus destructor* Thorne) disease (Gao et al., 2019). These challenges have resulted in yield declines and reduced crop quality, posing significant obstacles to the sustainable development of sweet potato cultivation.

Previous studies have demonstrated that fertilization plays a critical role in regulating soil enzymatic activity, increasing crop yields, and improving soil microecology (Plaza et al., 2004). However, inappropriate agricultural practices and excessive nitrogen fertilization can disrupt agroecosystem homeostasis, leading to the accumulation of fungal toxins, nitrosamines, and ammonia in the soil, which negatively affects soil enzyme activity (Barabasz et al., 2002). Błońska et al. (2017) reported that soil enzyme activity largely depends on organic nitrogen levels and mineral fertilizer application. Nitrogen is a key nutrient influencing sweet potato yield, photosynthetic efficiency, and the absorption of phosphorus and potassium. While appropriate nitrogen fertilization enhances yield (Tsuno and Fujise, 1963), excessive nitrogen application reduces the translocation of photosynthetic products to the storage roots, lowering the dry matter harvest index (Hartemink et al., 2000; Ankumah et al., 2003). Thus, optimizing nitrogen application rates is essential for maximizing sweet potato productivity (Solano et al., 2018; Krochmal-Marczak et al., 2018, 2020).

Additionally, nitrogen fertilization significantly influences soil microbial communities (Geisseler and Scow, 2014; Zhao et al., 2015). It can enhance microbial carbon source utilization and functional diversity, shaping the overall microbial community structure (Kavamura et al., 2018; Li et al., 2020; Cao et al., 2024). Nitrogen fertilizers and certain composts have also been shown to inhibit nematode hatching and growth (Liang and Chen, 2002). Long-term fertilization can reduce nematode populations and significantly suppress plant-parasitic nematodes in soil (Pan et al., 2010). However, limited information is available on how nitrogen fertilization affects soil microbial communities, enzyme activity, and stem nematode incidence in continuous sweet potato cropping systems.

Therefore, this study aimed to investigate the effects of different nitrogen application rates on sweet potato yield, microbial community structure, enzyme activities, and stem nematode incidence in a five-year continuous cropping system. We also assessed the role of nitrogen fertilization in shaping microbial diversity and reducing nematode damage. The findings from this study will provide valuable insights into the mechanisms underlying nitrogen-mediated regulation of soil microbial communities and nematode suppression, offering a scientific basis for optimizing nitrogen fertilizer application in sweet potato production.

## 2 Materials and methods

### 2.1 Study site

The study was conducted in an experimental field located at the Key Laboratory of Crop Stress Biology of Hebei Normal University of Science and Technology, Lulong County, Qinghuangdao City, Hebei Province, China (E118°96′, N39°95′). The annual average temperature is 10.7°C, the annual average precipitation is 725 mm and a frost-free period of 169 days. The soil is classified as brown soil with a pH (water) of 6.8 and an organic matter content of 11.3 g kg^−1^, available K at 0.79 g kg^−1^, available P at 21.6 mg kg^−1^ and available N at 54.0 mg kg^−1^. The experimental field has a five-year history of sweet potato planting and is known to be heavily infested with stem nematodes (Yang, 2023). The initial population density of nematodes per 100 grams of soil was described in Table 1 before planting sweet potatoes.

**Table 2:** Effects of nitrogen fertilization on population number of nematode in soil and stem nematode disease.

Treatment	Initial population (Individuals/100 g soil)	Final population (Individuals/100 g soil)	Reproduction factor (RF)	Disease incidence (%)
CK	56.67 ± 5.77b	984.33 ± 45.57a	17.47	55.00 ± 2.89a
N0	93.33 ± 15.27ab	683.33 ± 30.55c	7.43	30.00 ± 5.00b
N10	153.33 ± 20.82a	626.67 ± 20.82c	4.12	28.33 ± 3.33b
N20	60.00 ± 34.64b	760.00 ± 30.00b	16.71	41.67 ± 4.41b
N40	90.00 ± 60.83b	686.67 ± 15.28c	9.30	36.67 ± 4.41b

Means ± standard errors (*n* = 3) followed by different letters indicate significant differences between levels of a factor (within a column) according to Duncan^’^s multiple range test at *p* < 0.05.

### 2.2 Experimental materials

The stem nematode-susceptible sweet variety Tengfei used in this study, is one of the most important commercial cultivars widely planted in China. It was purchased from Zhongshu Agricultural Technology Co., Ltd. (Lulong, China). The experiment utilized urea (N 46%), calcium superphosphate (P_2_O_5_ 15%), and potassium sulfate (K_2_O 50%) as N, P, and K fertilizers, respectively. These chemical fertilizers were obtained from local agricultural material stores (Lulong, Hebei Province, China).

### 2.3 Experimental design

The experimental layout was a randomized complete block design with five treatments and three replicates per treatment. Each plot size was 8 m × 5 m. Five nitrogen fertilizer treatments at 72 kg (N0), 64.8 kg (N10), 57.6 kg (N20), 43.2 kg (N40) and 0 kg (CK) of urea-N per hectare were applied as basal fertilization at the start of the cropping season. In addition, all treatments received 66 kg P_2_O_5_ ha^−1^ (calcium superphosphate) and 156 kg K_2_O ha^−1^ (potassium sulfate) at the start of the cropping season. Fertilizers were applied by hand as a surface broadcast before sowing and incorporated into the 0–15 cm topsoil using a rotary tiller. Sweet potato seedlings, up to 15 cm in size, were selected and transplanted in mid-May, with a row spacing of 85 cm and a density of 67,500 plants ha^−1^. In all plots, visible weeds were manually removed during the growing season. Moreover, no plant protection from diseases and pests was applied because it was not necessary (no diseases and pests). Storage roots were harvested in the technical ripeness stage in early October.

### 2.4 Plant growth, disease index and soil sampling

The growth of sweet potato plants was investigated at the harvesting stage. Five plants per treatment from each replicate were dug up, and the vine length, number of branches, number and weight of storage roots, and fresh weight of vines and leaves per plant were measured and recorded. Storage roots were examined for lesions and assigned a disease severity rating on a scale of 0–4(0 = healthy; 1 ≤ 25% of the lesions areas of storage root; 2 = 25–50% of the lesions areas of storage root; 3 = 50–75% of the lesions areas of storage root; 4 = 75–100% of the lesions areas of storage root), the disease incidence was calculated using the formula provided below (Shi et al., 2024).


$$\begin{array}{l} \mathrm{Disease incidence}=(\mathrm{number of diseased sweet potato storage}\\ \mathrm{roots}/\mathrm{total number of sweet potato storage roots})\times 100\% \end{array}$$

Before crop harvest, five soil cores were randomly collected in each plot at 0–20 cm depth by a 2-cm-diameter soil auger (Zhao et al., 2014). Fresh soil samples in each plot were mixed to create a composite sample, sealed in plastic boxes, stored in an ice box, and transported to the laboratory. Visible plant debris was then carefully removed, after which the samples were divided into two parts. One subsample was stored at −80°C for extraction of soil DNA and subsequent molecular analysis, while the other subsample was stored at 4°C for the analysis of microbial population, Biolog, soil enzyme activities, and number of stem nematode individuals.

For the extraction of stem nematode, the method described by the Baermann funnel method (Jenkins, 1964; Botelho et al., 2019) was employed. Soil samples (100 g each) were added to the tissue paper and placed on the funnel, filtered as water was added over the soil, incubated for 48 h, and the nematodes in the flat-bottomed glass test tube were collected. The number of stem nematode individuals in each sample was determined under an inverted optical microscope. The reproduction factor (RF) of stem nematode in the study was determined using the formula RF=Pf/Pi where Pf was the final nematode population and Pi was the initial population density (Mwaura et al., 2017).

### 2.5 Soil microbial population determination

Culturable populations of soil microbes were determined using the method described by Zhang et al. (2014). The media used, and the target microbial populations were as follows: Luria-Bertani (LB) medium for bacteria, potato dextrose agar (PDA, Difco) medium for fungi, and Gause NO. 1 for actinobacteria. One gram of soil samples was transferred to a 15 mL Falcon tube, to which sterile distilled water was added to a total volume of 10 mL, and then shaken vigorously. Ten-fold serial dilutions were prepared, and 50 μL aliquots from each tube were spread on all three types of media. Each soil sample was assessed in triplicate. The inoculated plates were incubated at 28°C for varying durations (48 h for bacteria, 4 d for fungi, and 5 d for actinobacteria). The colonies that appeared on the media were counted and expressed as cfu/g dry soil, determined by weight after drying at 105°C to a constant weight.

### 2.6 Biolog analysis

BIOLOGTM ECO plates (Biolog Inc., Hayward, CA, USA), which contained three replicates of 31 carbon sources and a water blank containing no carbon source (Garland and Mills, 1991), were used to generate community-level physiological profiles for rhizosphere soil samples. Average well color development (AWCD), Shannon’s diversity index (*H*) and the substrate richness (*S*) were calculated following the methodology described previously (Zhu et al., 2013).

### 2.7 Determination of the soil enzyme activities

Soil urease and invertase activities were determined according to the method described by Guan (1986). For urease activity, 5 g of soil was added 1 mL of methylbenzene, and after 15 min, the sample was mixed with 10 mL of 10% urea solution and 20 mL of citrate buffer (pH 6.7). The mixture was filtered after incubation at 37°C for 24 h. The formation of ammonium was measured spectrophotometrically at 578 nm. Soil invertase activity was determined by incubating 5 g of soil with 15 mL of an 8% sucrose solution at 37°C for 24 h. The suspension reacted with 3,5-dinitrosalicylic acid, and the absorbance was measured at 508 nm. Soil phosphatase activity was measured using p-nitrophenyl phosphate as a substrate (Tabatabai and Bremner, 1969). The soil invertase, urease, and phosphatase activities are expressed as mg of glucose, NH_3_-N, and phenol released per 1 g of dry soil per 24 h, respectively.

### 2.8 Illumina MiSeq sequencing and analysis

According to the protocol, the total soil DNA was extracted using the Fast Soil DNA kit (Omega Biotek, Inc.). The concentration and purity of the extracted DNA were determined using a spectrophotometer, and the DNA was stored at −20°C for subsequent experiments. PCR amplification was performed using tagged universal bacterial and fungal primer pairs. For bacteria, the V3-V4 regions of the 16S rRNA genes were amplified using the primers 338F (5′-ACTCCTACGGGAGGGAGGA-3′) and 806R (5′-GGACTACHVGGGTWTCTAAT-3′) (Lu et al., 2018). For fungi, the internal transcribed spacer regions were amplified with primers ITS1F (5′-CTTGGTCATTTAGAGGAAGTAA-3′) and ITS2R (5′-GCTGCGTTCTTCATCGATGC-3′) (Zhang et al., 2020). The PCRs were performed using the following protocol: denaturation at 95°C for 3 min, followed by 27 cycles of 95°C for 30 s, 55°C for 30 s, and 72°C for 45 s, then a final extension at 72°C for 10 min (PCR instrument: ABI GeneAmp^®^ 9700). The PCR products were purified using 2% agarose gel and the AxyPrep DNA Gel Extraction Kit (Axygen Biosciences, Union City, CA, USA). Purified amplicons were sequenced in a paired end format using the Illumina MiSeq platform by Majorbio BioPharm Technology Co. Ltd. (Shanghai, China). Then, the 16S and ITS sequences of the high-quality paired-end reads were merged by FLASH software (Magoc and Salzberg, 2011), and the barcodes of the final sequences were filtered and removed by Mothur (Version 1.30.2) (Kozich et al., 2013).^1^ The operational taxonomic units (OTUs) were clustered at 97% similarity based on the merged sequences using the UPARSE pipeline (Edgar, 2013). Using the Ribosomal Database Project (RDP) Classifier tool, the 16S rRNA gene sequences of bacteria were matched with those sequences stored in the SILVA Database, using a confidence threshold of 70% (Amato et al., 2013). The ITS gene sequences of fungi were classified against the UNITE ITS database using the k-nearest neighbor algorithm (Abarenkov et al., 2010). All raw sequencing data were deposited in the National Center for Biotechnology Information (NCBI) under accessed numbers PRJNA1112307 and PRJNA1119064.

### 2.9 Statistical analysis

Data analyses were performed using the SAS statistical software (Version 9.21, SAS Institute Inc., USA). Differences in sweet potato growth parameters, nematode population, enzymatic activity, and microbial characteristics among the treatments were assessed by One-way Analysis of Variance (ANOVA). The results in the table are presented as mean ± standard deviation. Significant differences between treatments were assessed using Duncan’s multiple range test with a significance level of *p* < 0.05.

The OTUs abundance was normalized according to the sample with the least sequences, and all subsequent analyses were performed according to the normalized data. Chao richness and Shannon diversity were calculated for bacterial and fungal’s alpha-diversity assessment, and treatment effect on alpha-diversity was also examined by an ANOVA. Non-metric multidimensional scaling (NMDS) was conducted to visualize bacterial and fungal community composition based on a Bray–Curtis dissimilarity matrix. The significance was tested by analysis of similarities (ANOSIM). Community structure was compared through principal coordinate analysis (PCoA) utilizing unweighted UniFrac distance metrics. These analyses were calculated with QIIME (Version 1.9.1)^2^ and visualized using R Software (Version 2.15.3). The heatmap figure was generated using custom R scripts. All bioinformatics analyses were conducted using the online tools of Majorbio Cloud Platform.^3^

## 3 Results

### 3.1 Effects of nitrogen fertilization on growth of sweet potato and stem nematode disease

No significant differences in branch number, number of storage roots, or fresh weight of vines and leaves per plant were observed between nitrogen fertilization treatments and CK at *p* < 0.05 level at harvesting time (Table 1). However, the N10 treatment significantly increased vine length and sweet potato yield (*p* < 0.05). Nitrogen fertilization significantly reduced the incidence of stem nematode in sweet potato compared to CK. Among the nitrogen fertilization treatment, the N10 treatment reduced the nematode disease index from 55.0 to 28.33%, while the final population number of nematodes extracted from the N10 treatment was significantly lesser than other treatments (Table 2).

**Table 1:** Effects of nitrogen fertilization on sweet potato growth and yield.

Treatment	Vine length (cm)	Number of branches	Number of storage roots per plant	Fresh weight of vines and leaves per plant (kg)	Yields (tonne·ha^−1^)
CK	182.70 ± 20.54b	4.00 ± 0.35a	2.06 ± 0.18a	0.44 ± 0.15a	28.12 ± 1.61b
N0	209.00 ± 14.80ab	4.10 ± 0.27a	2.23 ± 0.23a	0.32 ± 0.02a	30.13 ± 0.92ab
N10	232.00 ± 8.33a	5.00 ± 0.46a	2.26 ± 0.09a	0.45 ± 0.04a	30.23 ± 0.79a
N20	186.00 ± 16.29ab	4.30 ± 0.07a	2.03 ± 0.30a	0.41 ± 0.06a	29.65 ± 1.09ab
N40	174.30 ± 1.45b	4.30 ± 0.48a	2.43 ± 0.18a	0.45 ± 0.12a	28.61 ± 0.57ab

Means ± standard errors (*n* = 3) followed by different letters indicate significant differences between levels of a factor (within a column) according to Duncan^’^s multiple range test at *p* < 0.05.

### 3.2 Effects of nitrogen fertilization on soil enzyme activity

**Figure 1:**
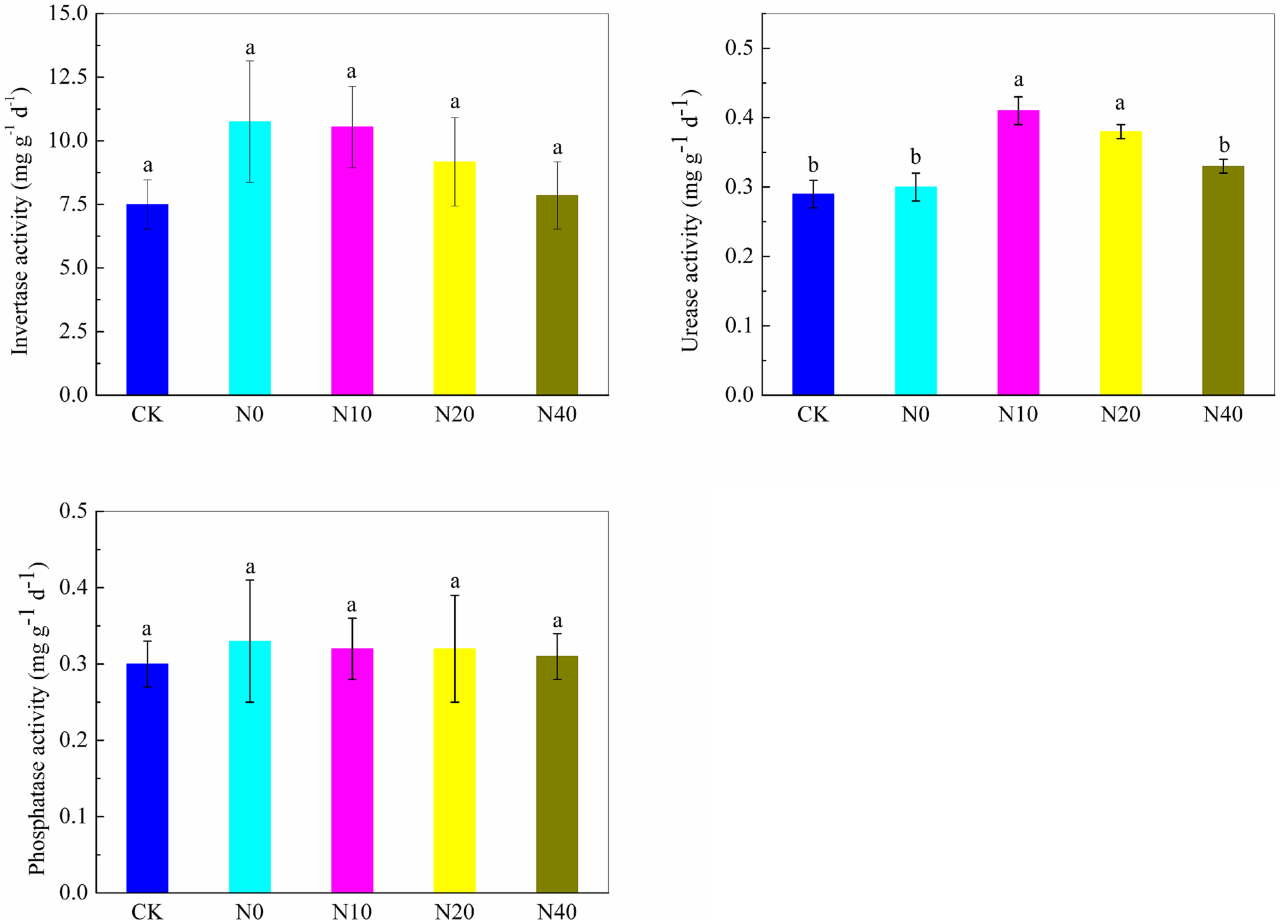
Soil enzyme activity under different nitrogen fertilization treatments.

The responses of soil enzyme activities, including invertase, urease, and phosphatase, under nitrogen fertilization, are presented in Figure 1. Overall, as the nitrogen fertilization dose decreased, the activities of the three tested soil enzymes initially increased and then decreased. No significant differences in invertase and phosphatase activities were observed among the treatments. However, the N10 and N20 treatments caused significant differences in urease activity. The N10 and N20 treatments noticeably enhanced urease activity by 41.37 and 31.03%, respectively, compared to the CK treatment. These results suggest that nitrogen fertilization influences soil urease, significantly altering the enzyme activity in sweet potato soil, with the N10 treatment having the most pronounced effect.

### 3.3 Effects of nitrogen fertilization on soil microbial population

**Figure 2:**
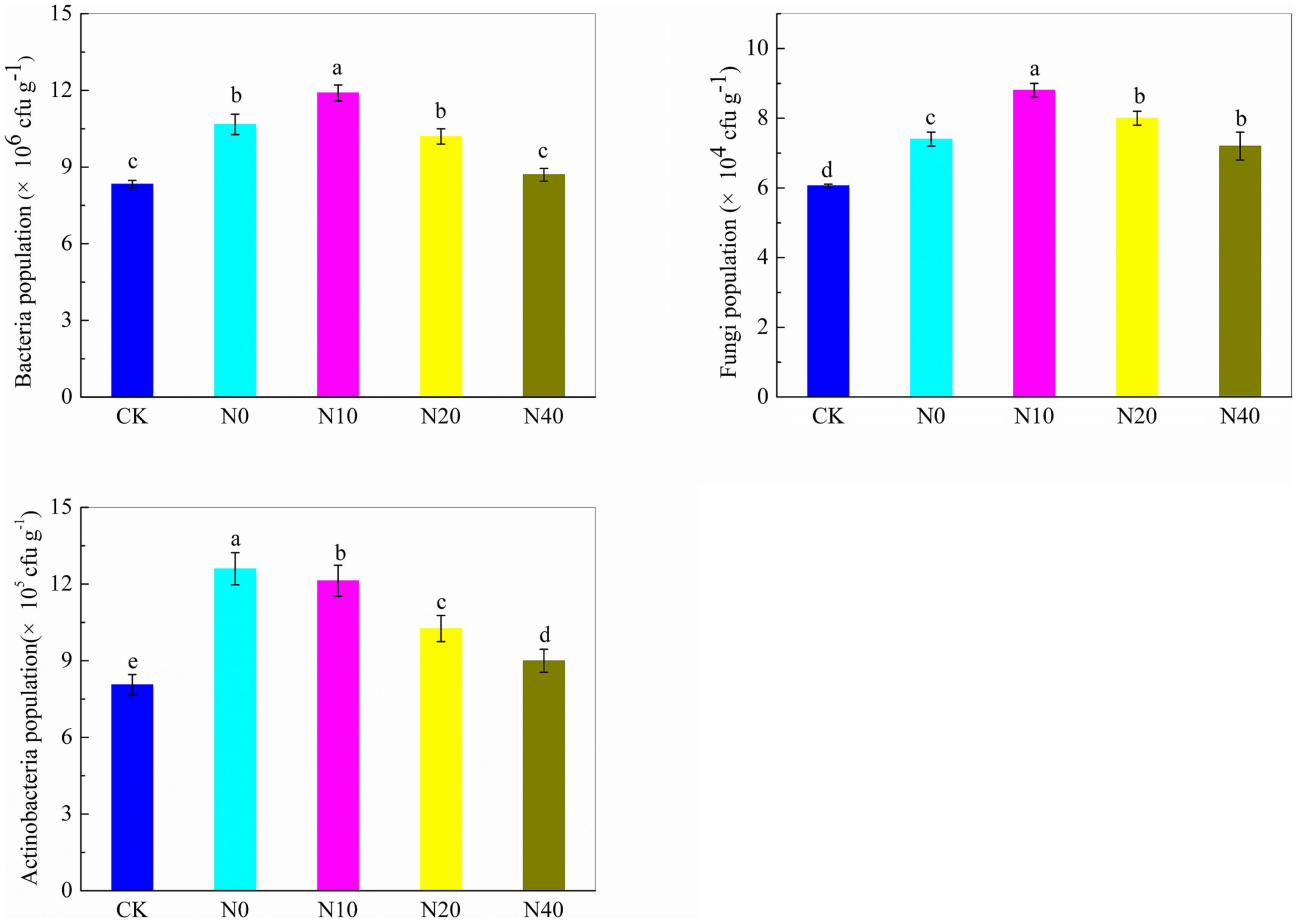
Soil microbial population under different nitrogen fertilization treatments.

Figure 2 illustrates the effects of nitrogen fertilization on the population of soil bacteria, fungi, and actinobacteria. In general, nitrogen fertilization at all experimental doses promoted the growth of soil bacteria, fungi, and actinobacteria. For bacteria, population numbers in the N0, N10, and N20 treatments were higher than the N40 and CK treatments. No significant differences in bacterial numbers were observed between the N40 and CK treatments. Among the nitrogen fertilization treatments, N10 had a significantly higher CFU count (*p* < 0.05) than other treatments and CK. Moreover, the N10 treatment increased the bacterial population by 42.8% compared to the CK treatment. The fungal population increased across all nitrogen fertilization treatments, with significant differences observed, particularly in the N10 treatment. The actinomycete population exhibited similar behavior to the fungal population. The actinomycete population in nitrogen fertilization treatments was higher than in the CK treatment. The N0 and N10 treatments significantly increased the actinomycete population compared to the N20, N40, and CK treatments.

### 3.4 Carbon utilization capacity of the microbial community

**Figure 3:**
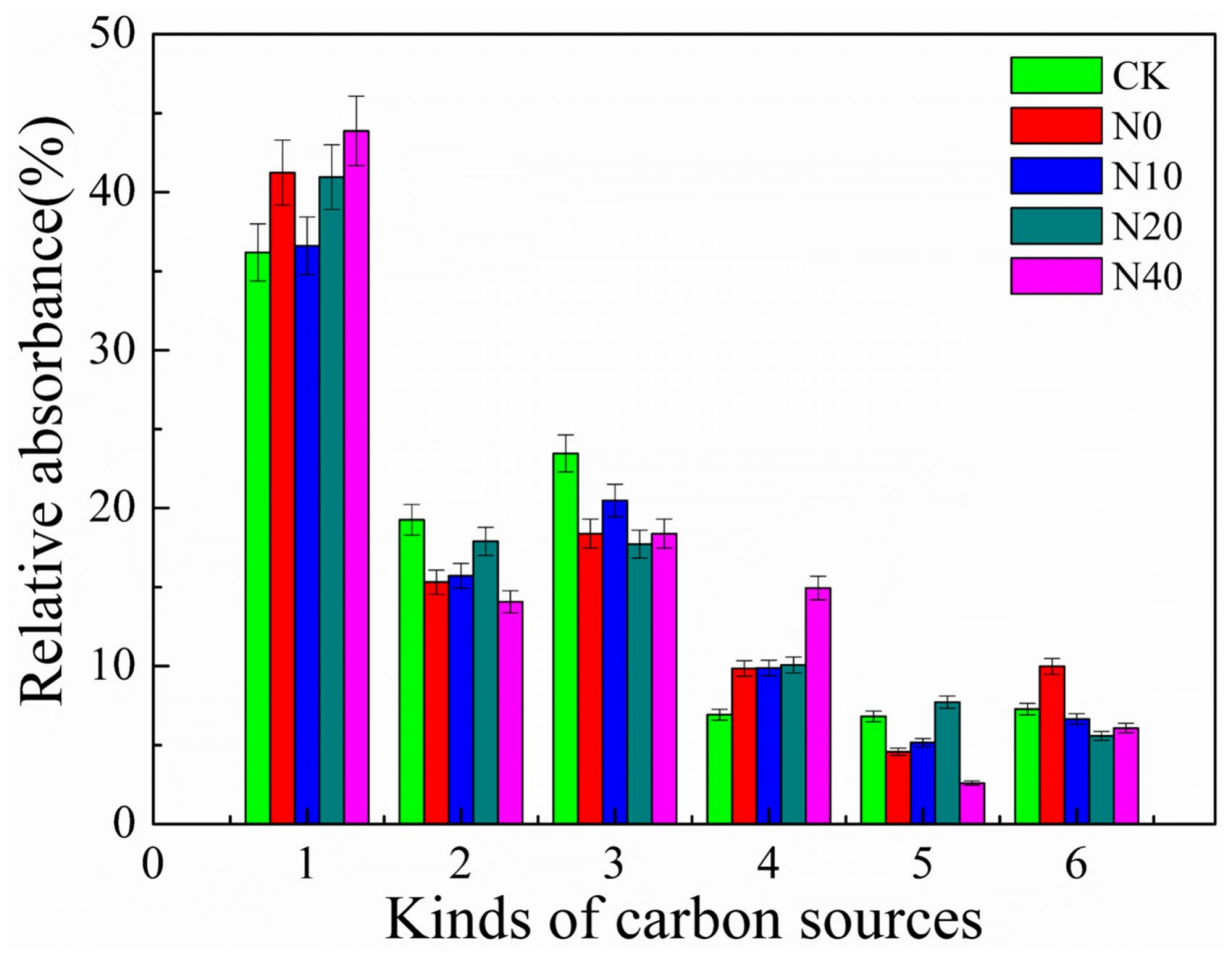
Relative utilization ratios of the six kinds of carbon sources in Biolog EcoPlates, including Carbohydrates (1), Carboxylic acids (2), Amino acids (3), Polymers (4), Aromatic compounds (5), and Amines (6), were assessed by the rhizosphere microbial community across different treatments.

**Figure 4:**
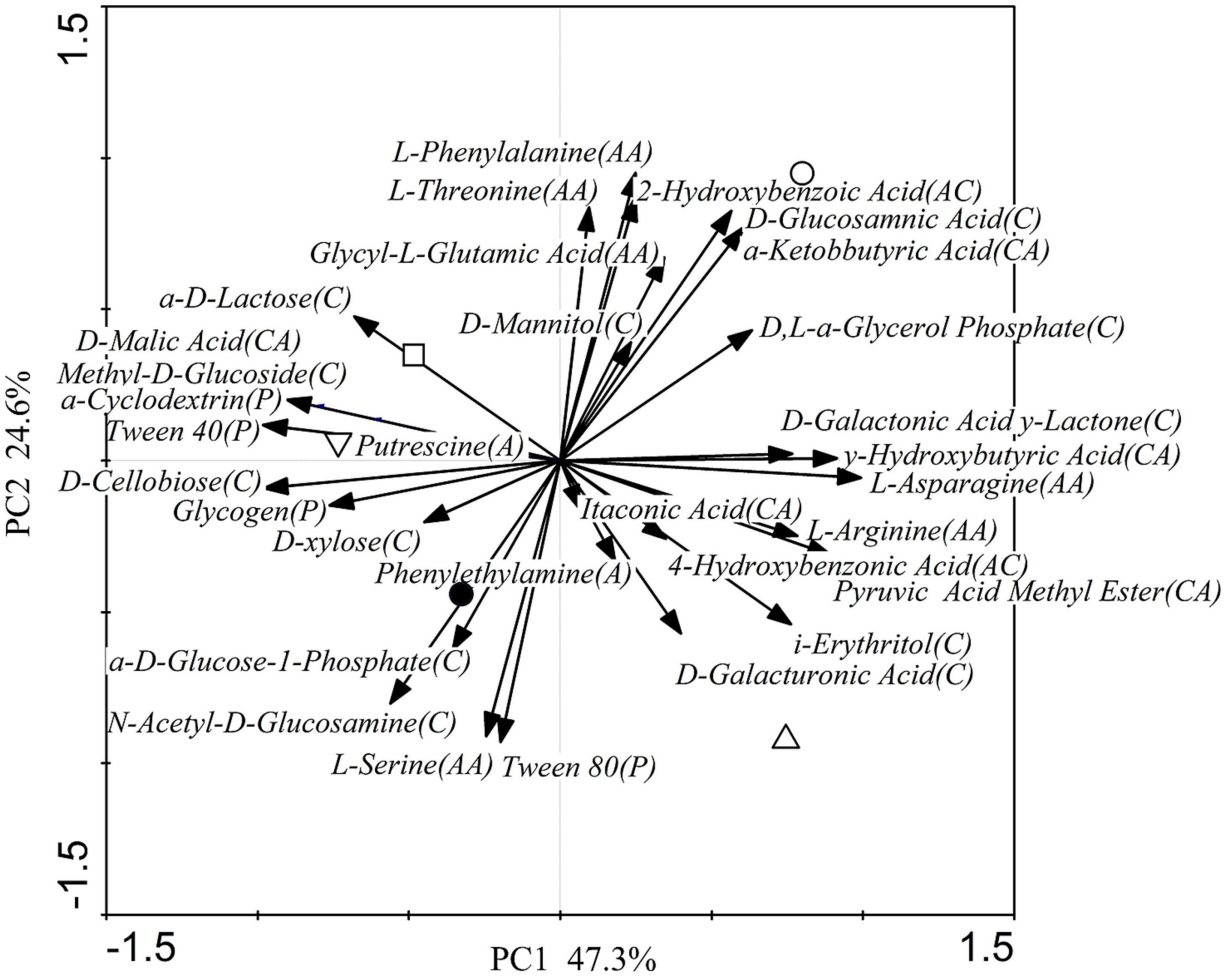
Principal component analysis (PCA) was conducted on the catabolic profiles of the rhizosphere microbial community in CK (○), N0 (●), N10 (△), N20 (□) and N40 (▽) treatments.

**Figure 5:**
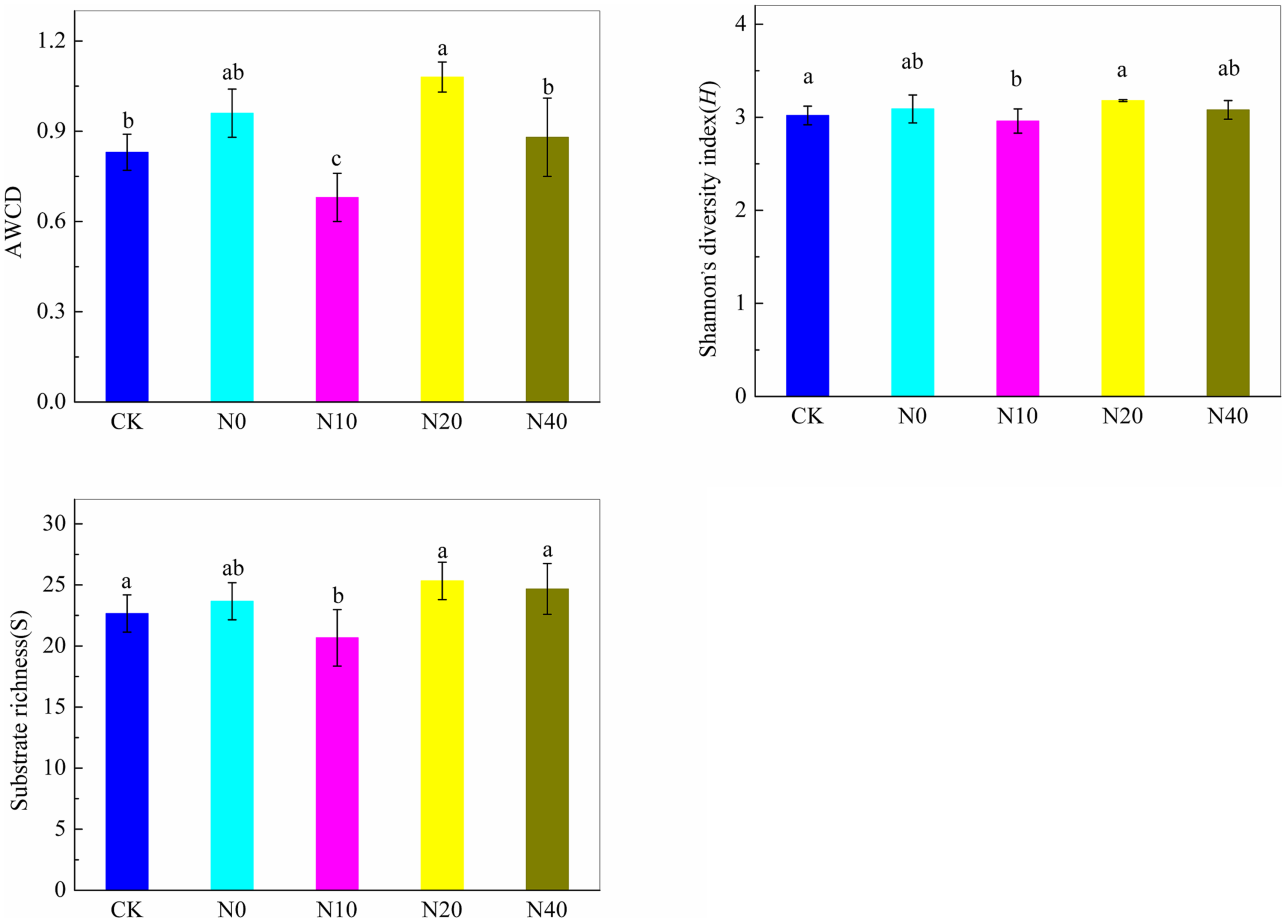
Catabolic diversity for the rhizosphere microbial communities across different treatments.

The Biolog EcoPlates comprised 31 carbon sources, including nine carboxylic acids, six amino acids, four polymers, two amines, eight carbohydrates, and two aromatic compounds. Figure 3 illustrates the relative utilization efficiency of the six types of carbon sources by the rhizosphere microbial community. Carbohydrates, amino acids, carboxylic acids, and polymers exhibited a high relative utilization efficiency. Conversely, aromatic compounds and amines demonstrated a low relative utilization efficiency. Compared to the control group (CK), obvious utilization differences were observed among the six types of carbon sources. These findings suggest that nitrogen fertilization can influence carbon utilization and metabolism by microbes in the rhizosphere soil. Principal component analysis (PCA) was performed to understand the variations in carbon utilization by rhizosphere microbes. Figure 4 shows the PCA results of the carbon substrate utilization profiles of the rhizosphere microbial community, with PC1 accounting for 47.3% and PC2 accounting for 24.6% of the data variability. The catabolic profiles of the rhizosphere microbial community displayed distinct differences between the CK and the nitrogen fertilization treatments, indicating a marked divergence in the rhizosphere microbial community structure. However, the communities from the N0, N20, and N40 treatments exhibited closer proximity in quadrant II and III. Additionally, significant differences (*p* < 0.05) were observed between the N10 and CK treatments in terms of average well color development (AWCD), Shannon’s diversity index, and substrate richness (Figure 5).

### 3.5 Alpha diversity of microbial community

For bacteria, a total of 593,655 effective sequences with an average length of 415 bp and about 39,577 sequences per sample (49,721–57,640 sequences) were obtained. The number of OTUs ranged from 3,486 to 4,042 at a 97% similarity. For fungi, a total of 689,880 effective sequences with an average length of 233 bp and about 45,992 sequences per sample (46,143–55,111 sequences) were obtained. The number of OTUs ranged from 414 to 518 at a 97% similarity. The Good’s coverage values fluctuated between 96.7 and 99.8%, indicating that the sequencing depth was sufficient to capture diversity. The alpha diversity index (Table 3) showed that all diversity indices were affected to varying extents by the different treatments. The ACE and Chao1 indices represent community richness, while the Shannon and Simpson indices reflect community diversity and evenness, respectively. Compared to the CK treatment, the Chao1 and ACE indices significantly decreased (*p* < 0.05) following N0, N10, and N20 treatments, while the N40 treatment showed no significant difference, indicating that N0, N10, and N20 significantly affected soil bacterial richness. Additionally, significant differences in the Shannon and Simpson indices were observed between the N10 and CK treatments, indicating that the N10 treatment had a substantial effect on soil bacterial diversity. No significant differences in the Chao1 and ACE indices were detected among the nitrogen fertilization and CK treatments for fungal communities. However, nitrogen fertilization significantly reduced the fungal Simpson index compared to the CK treatment. The Shannon index was significantly higher in all nitrogen fertilization treatments than the CK treatment. The Sobs index was significantly higher in all nitrogen fertilization treatments compared to the CK treatment. Compared to the CK treatment, nitrogen fertilization significantly reduced the bacterial Pielou index and increased the fungi Pielou index. These results demonstrate that nitrogen fertilization significantly impacts the soil ecological environment.

**Table 3:** Alpha diversity of the bacterial and fungal communities under different treatments.

Microbial community	Treatment	Sobs	Chao1	ACE	Simpson	Shannon	Coverage	Pielou
Bacteria	CK	3,384 ± 91.3a	4,895 ± 109.0a	5,026 ± 123.8a	0.0044 ± 0.0002b	6.85 ± 0.02a	0.967	0.8294 ± 0.0013a
	N0	3,566 ± 100.2b	4,530 ± 187.4b	4,621 ± 187.8b	0.0052 ± 0.0003ab	6.68 ± 0.03b	0.970	0.8148 ± 0.0011c
	N10	3,556 ± 66.2b	4,487 ± 120.6b	4,619 ± 90.1b	0.0058 ± 0.0007a	6.65 ± 0.07b	0.969	0.8113 ± 0.0040c
	N20	3,599 ± 69.9b	4,521 ± 124.8b	4,634 ± 137.9b	0.0052 ± 0.0004ab	6.70 ± 0.04b	0.970	0.8218 ± 0.0005b
	N40	3,744 ± 74.6c	4,771 ± 107.0a	4,849 ± 94.0a	0.0048 ± 0.0006b	6.78 ± 0.07a	0.968	0.8277 ± 0.0009ab
Fungi	CK	424 ± 9.1a	481.9 ± 30.3a	478.0 ± 18.3a	0.2775 ± 0.0125a	2.68 ± 0.06c	0.998	0.4390 ± 0.0069c
	N0	475.3 ± 44.4b	517.5 ± 52.2a	519.6 ± 54.4a	0.1243 ± 0.0375c	3.48 ± 0.24a	0.998	0.5827 ± 0.0191a
	N10	448.8 ± 19.3b	499.2 ± 25.4a	499.3 ± 30.6a	0.1855 ± 0.0315b	3.14 ± 0.16b	0.998	0.5110 ± 0.0085b
	N20	465 ± 11.7b	521.4 ± 31.9a	517.4 ± 28.7a	0.1198 ± 0.0182c	3.46 ± 0.14a	0.998	0.5747 ± 0.0174a
	N40	447.7 ± 27.6b	506.9 ± 40.6a	503.6 ± 37.5a	0.1860 ± 0.0313b	3.10 ± 0.16b	0.998	0.5018 ± 0.0159b

Data were calculated from three replicates per treatment and are presented as mean ± standard deviation. Different letters indicate significant differences according to Fisher’s least significant difference and Duncan’s multiple range tests (*p* < 0.05).

**Figure 6:**
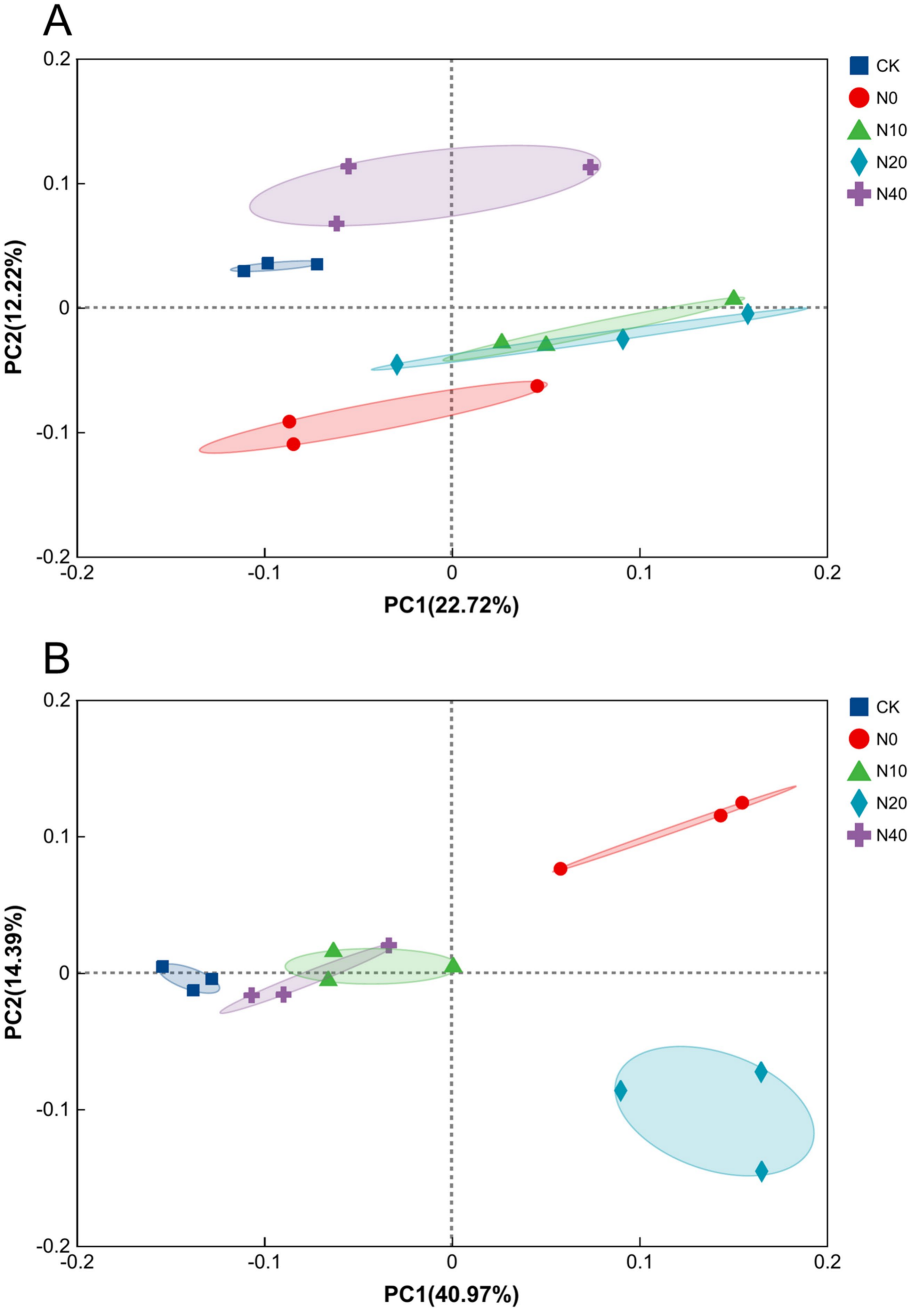
Principal coordinate analysis (PCoA) of bacterial **(A)** and fungal **(B)** community structure based on the number of detected OTUs from 15 samples (*R* = 0.84, *p* = 0.001).

**Figure 7:**
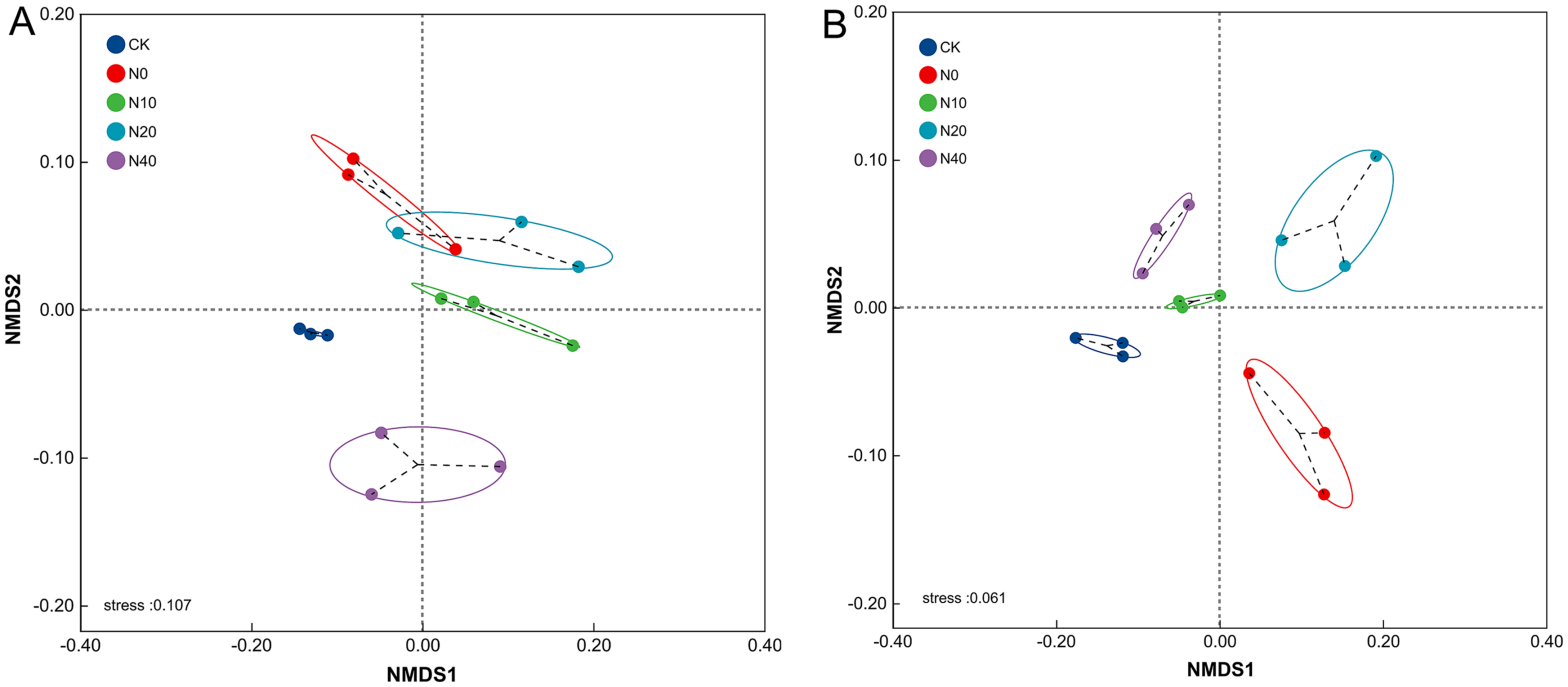
Non-metric multidimensional scaling (NMDS) ordination illustrating compositional differences in bacterial **(A)** and fungal **(B)** communities associated with soils with different nitrogen fertilization treatments.

Additionally, to further illustrate the differences between samples, PCoA analysis (Figure 6) was performed to visualize the effects of nitrogen fertilization on soil microbial community structure. The results showed that bacterial communities from the N0, N10, and N20 treatments differed significantly from those of the CK treatment, indicating that nitrogen fertilization significantly altered the soil bacterial community structure. Although the N10 treatment showed a smaller distance from the CK treatment, a similar trend was observed in the fungal community structure. Non-metric multidimensional scaling (NMDS) of Bray-Curtis dissimilarities also showed that microbial communities in the N10 treatment were different from those in the other treatments (Figure 7). ANOSIM confirmed significant separation of the bacterial (*R* = 0.74, *p* = 0.001) and fungal (*R* = 0.86, *p* = 0.001) communities, indicating a shift in microbial community composition. Overall, the results revealed significant differences in microbial community structures between the nitrogen fertilization and CK treatments.

### 3.6 Structures of the microbial community

**Figure 8:**
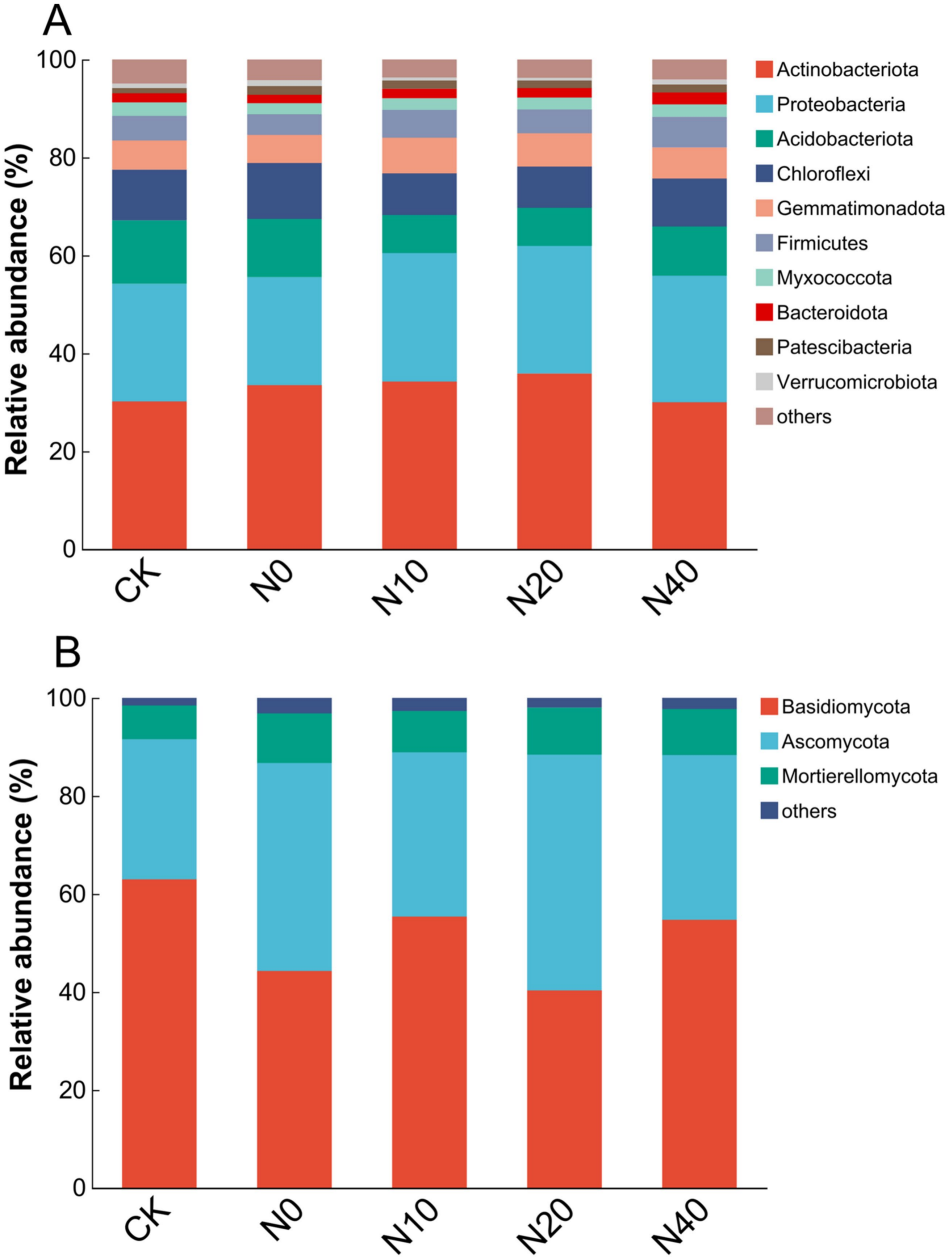
Comparison of bacterial **(A)** and fungal **(B)** communities at the phylum level. Relative read abundances of different bacterial **(A)** and fungal **(B)** phyla within the different communities are shown. Sequences that could not be classified into any known group are labeled as “Other.”

**Figure 9:**
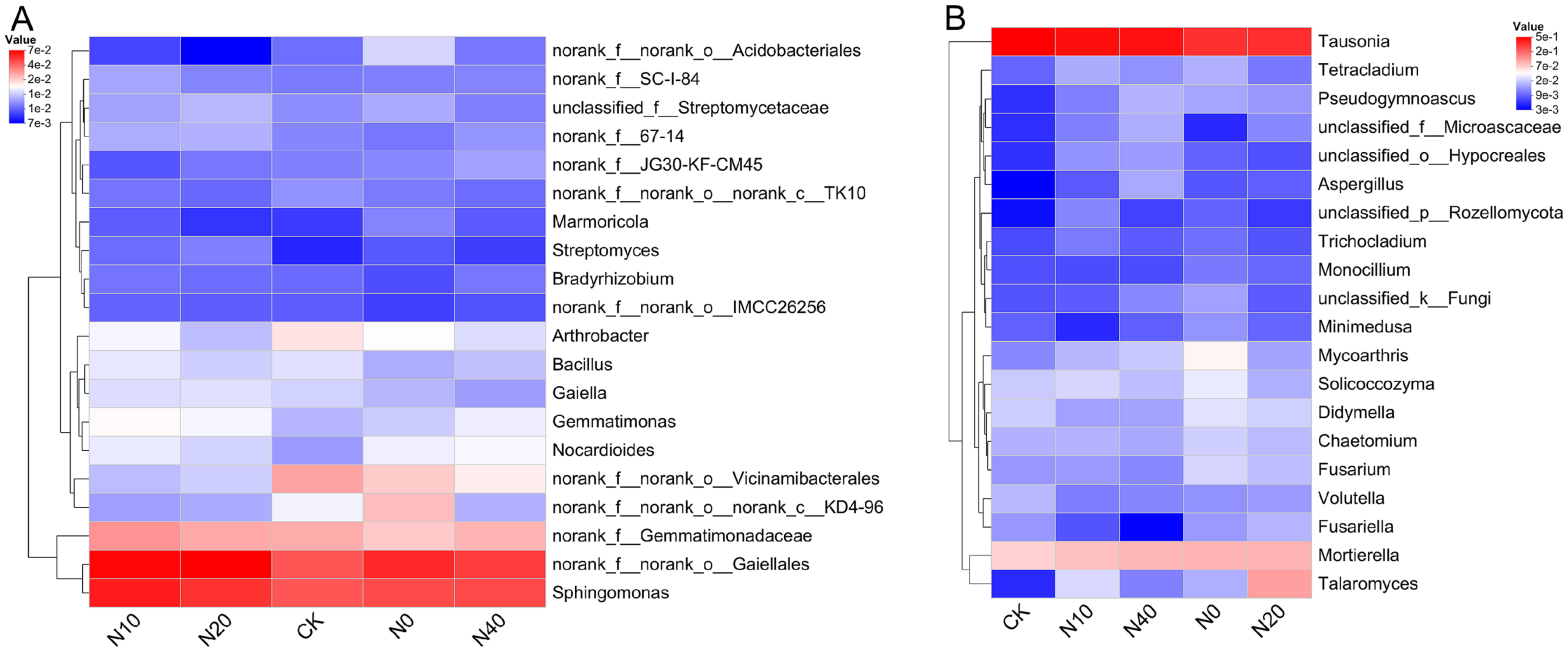
Heatmap of bacterial **(A)** and fungal **(B)** communities based on the 20 most abundant genera in each treatment. Green and red represent low and high relative abundances, respectively.

The compositions of bacterial and fungal communities were significantly influenced by the different nitrogen fertilization treatments (Figure 8). Ten bacterial phyla were identified across all soil samples from the different treatments (Figure 8A). The dominant bacterial phyla were *Actinobacteriota* (29.98–35.86%), *Proteobacteria* (22.05–26.28%), *Acidobacteriota* (7.74–12.96%), *Chloroflexi* (8.36–11.40%), *Gemmatimonadota* (5.76–7.30%), and *Firmicutes* (4.21–6.24%), with minor phyla including *Myxococcota* (2.25–2.75%), *Bacteroidota* (1.70–2.46%), *Patescibacteria* (1.04–1.82%), and *Verrucomicrobiota* (0.55–1.14%). Compared to the CK treatment, the N10 treatment increased the relative abundance of *Actinobacteriota*, while *Acidobacteriota* abundance decreased under the N10 and N20 treatments (*p* < 0.05). The effects of different treatments on the dominant bacteria genera (top 20) in rhizosphere soil are shown in Figure 9A. According to the heatmap, the relative abundances of *Sphingomonas, Nocardioides, Gemmatimonas,* and *Streptomyces* significantly increased in all nitrogen fertilization treatments compared to the CK treatment. Specifically, *Sphingomonas* and *Gemmatimonas* were most abundant in the N10 treatment. However, nitrogen fertilization did not alter the *Bacillus* population. Additionally, the relative abundance of *Arthrobacter* decreased in all nitrogen fertilization treatments.

For the fungal community, *Basidiomycota* (40.31–62.96%) was the most dominant phylum across treatments, followed by *Ascomycota* (28.61–48.11%). The other fungal phylum identified was *Mortierellomycota* (6.89–10.11%) (Figure 8B). Specifically, the relative abundance of *Basidiomycota* was lower in nitrogen fertilization treatments compared to the CK treatment. At the same time, *Ascomycota* was more abundant under nitrogen fertilization. At the genus level, 20 genera were present in the soil samples of all treatments (Figure 9B). The relative abundances of *Mortierrella* and *Mycoarthris* significantly increased under all nitrogen fertilization treatments, while *Volutella* was more abundant in the CK treatment.

## 4 Discussion

Determining the optimal nitrogen fertilization strategy is essential for maximizing crop yields while minimizing environmental risks. Crop yield is a commonly used indicator for determining optimal nitrogen levels (Wang et al., 2017). In this study, no significant differences in sweet potato yield were observed among the nitrogen fertilization treatments. However, the highest yield was recorded in the N10 treatment. Moreover, the N10 treatment significantly reduced the incidence of stem nematode disease, lowering its occurrence from 55.0 to 28.33%. This finding aligns with previous studies indicating that nitrogen fertilization can mitigate nematode infections (Oka and Yermiyahu, 2002; Oka and Pivonia, 2003; Barker et al., 1971). Furthermore, nitrogen fertilization influenced the reproduction factor of stem nematodes in the soil, consistent with Shakeel et al. (2022), who reported that different nitrogen levels significantly affected nematode reproduction. The inhibitory effect of nitrogen fertilization on nematodes is attributed to urea hydrolysis by soil urease, producing ammonia, which is detrimental to nematode survival (Tenuta and Lazarovits, 2002; Oka and Pivonia, 2002; Wang et al., 2010). In contrast, the N0 treatment did not significantly increase yield, reinforcing previous findings that beyond a certain threshold, additional nitrogen application does not enhance crop productivity (Liu and Wiatrak, 2012; Qiao et al., 2012; Li et al., 2019). These results suggest that N10 is the most effective nitrogen fertilization strategy for promoting sweet potato yield while reducing stem nematode incidence.

Soil enzymes are crucial for biochemical processes and nutrient cycling, playing an essential role in maintaining soil fertility (Liu et al., 2008). Urease facilitates urea hydrolysis into carbon dioxide and ammonia, influencing nitrogen uptake by crops. Phosphatase releases inorganic phosphorus from organic matter, making it available to plants (Burns et al., 2013), while invertase hydrolyzes sucrose into glucose and fructose, contributing to the carbon cycle (Zhang et al., 2004; Gianfreda et al., 2005). In our study, urease and invertase activities increased with nitrogen application, consistent with previous findings (Wang et al., 2008; Liang et al., 2014). However, excessive nitrogen application suppressed urease activity, as also observed by Sun et al. (2020). No significant differences were detected in phosphatase activity among treatments, in contrast to Allison et al. (2006), who reported increased phosphatase activity with nitrogen fertilization. These discrepancies may be due to variations in soil type and properties. Appropriate nitrogen management strategies enhance soil enzyme activity, promoting organic matter mineralization, urea decomposition, and nutrient cycling. When indigenous soil microorganisms have obtained sufficient nitrogen, carbon sources, and a favorable ecological environment, their reproduction and metabolic activities accelerate, enhancing nutrient absorption and utilization by plants (Gu et al., 2021). In our study, urease activity and microbial abundance were particularly responsive to nitrogen application, with the N10 treatment significantly enhancing both. As urea is rapidly converted to ammonia by soil urease, nitrogen fertilization may contribute to nematode suppression, supporting previous findings that ammonia-releasing fertilizers control plant-parasitic nematodes (Tenuta and Lazarovits, 2002).

The N10 treatment significantly influenced soil microbial diversity, as confirmed by Biolog and PCoA analyses. Previous studies have shown that organic and inorganic fertilizers impact soil microbial communities (Zhong et al., 2007; Gautam et al., 2020). At the genus level, *Sphingomonas*, *Gemmatimonas*, and *Nocardioides* were significantly more abundant in nitrogen-treated soils than in the CK treatment, with *Sphingomonas* and *Gemmatimonas* peaking in N10. *Basidiomycota* and *Ascomycota* were the dominant fungal phyla, with *Mortierella* and *Mycoarthris* increasing following nitrogen fertilization. *Sphingomonas* is known for its biocontrol properties, suppressing plant diseases through resource competition (Sanguin et al., 2009; Innerebner et al., 2011; Van Bruggen et al., 2014; Yu et al., 2019) and producing plant growth-promoting compounds (Enya et al., 2007). *Gemmatimonas* is more prevalent in healthy plants, suggesting a role in pathogen suppression (Yin et al., 2013). *Nocardioides* protects plants from ethylene-induced stress (Zhao et al., 2019), while increased bacterial richness and diversity, as demonstrated by Xiong et al. (2017), may enhance plant disease resistance. Additionally, *Mortierella*, a member of *Zygomycota*, stimulates microorganisms that suppress vanilla wilt disease via antibiotic production. Collectively, these findings suggest that the N10 treatment promotes beneficial microbes in sweet potato soil, contributing to reduced stem nematode incidence.

## 5 Conclusion

This study demonstrated that nitrogen fertilization significantly influences soil microbial community structure and enzymatic activities in continuous-cropping sweet potato fields, highlighting its role in shaping the microbiome and soil health. The application of N10 (64.8 kg ha^−1^) notably enriched beneficial microbial populations, particularly those associated with plant growth promotion and disease suppression, while enhancing soil urease activity and reducing stem nematode disease incidence. These microbiome shifts likely contribute to improved nutrient cycling, pathogen suppression, and overall soil resilience. Compared to CK and other nitrogen treatments, N10 optimized the microbial balance necessary for maintaining high yields and suppressing nematode infection. These findings underscore the critical role of nitrogen-mediated microbial modulation in sustainable disease management and agro-ecosystem stability. Judicious nitrogen application, particularly at optimal rates, can serve as a microbiome-based strategy to enhance soil health and crop productivity in continuous-cropping systems.

## Data Availability

The datasets presented in this study can be found in online repositories. The names of the repository/repositories and accession number(s) can be found in the article/Supplementary material.

## Footnotes

^1^ https://www.mothur.org/

^2^ http://qiime.org/

^3^ https://cloud.majorbio.com/page/tools.html
